# Seasonal variability and vertical distribution of autotrophic and heterotrophic picoplankton in the Central Red Sea

**DOI:** 10.7717/peerj.8612

**Published:** 2020-02-24

**Authors:** Najwa Al-Otaibi, Tamara M. Huete-Stauffer, Maria Ll. Calleja, Xabier Irigoien, Xosé Anxelu G. Morán

**Affiliations:** 1 Red Sea Research Center (RSRC), Division of Biological and Environmental Sciences and Engineering, King Abdullah University of Science and Technology (KAUST), Thuwal, Saudi Arabia; 2 Department of Climate Geochemistry, Max Planck Institute for Chemistry (MPIC), Mainz, Germany; 3 AZTI - Marine Research, Pasaia, Spain; 4 Basque Foundation for Science, IKERBASQUE, Bilbao, Spain

**Keywords:** Red Sea, Picoplankton, *Prochlorococcus*, *Synechococcus*, Picoeukaryotes, Heterotrophic bacteria, High nucleic acid, Low nucleic acid, Flow cytometry

## Abstract

The Red Sea is characterized by higher temperatures and salinities than other oligotrophic tropical regions. Here, we investigated the vertical and seasonal variations in the abundance and biomass of autotrophic and heterotrophic picoplankton. Using flow cytometry, we consistently observed five groups of autotrophs (*Prochlorococcus*, two populations of *Synechococcus* separated by their relative phycoerythrin fluorescence, low (LF-Syn) and high (HF-Syn), and two differently-sized groups of picoeukaryotes, small (Speuk) and large (Lpeuk)) and two groups of heterotrophic prokaryotes of low and high nucleic acid content (LNA and HNA, respectively). Samples were collected in 15 surveys conducted from 2015 to 2017 at a 700-m depth station in the central Red Sea. Surface temperature ranged from 24.6 to 32.6 °C with a constant value of 21.7 °C below 200 m. Integrated (0–100 m) chlorophyll *a* concentrations were low, with maximum values in fall (24.0 ± 2.7 mg m^−2^) and minima in spring and summer (16.1 ± 1.9 and 1.1 mg m^−2^, respectively). Picoplankton abundance was generally lower than in other tropical environments. Vertical distributions differed for each group, with *Synechococcus* and LNA prokaryotes more abundant at the surface while *Prochlorococcus*, picoeukaryotes and HNA prokaryotes peaked at the deep chlorophyll maximum, located between 40 and 76 m. Surface to 100 m depth-weighted abundances exhibited clear seasonal patterns for *Prochlorococcus,* with maxima in summer (7.83 × 10^4^ cells mL^−1^, July 2015) and minima in winter (1.39 × 10^4^ cells mL^−1^, January 2015). LF-Syn (0.32 – 2.70 × 10^4^ cells mL^−1^ ), HF-Syn (1.11 – 3.20 × 10^4^ cells mL^−1^) and Speuk (0.99 – 4.81 × 10^2^ cells mL^−1^) showed an inverse pattern to *Prochlorococcus,* while Lpeuk (0.16 – 7.05 × 10^4^ cells mL^−1^) peaked in fall. *Synechococcus* unexpectedly outnumbered *Prochlorococcus* in winter and at the end of fall. The seasonality of heterotrophic prokaryotes (2.29 – 4.21×10^5^ cells mL^−1^ ) was less noticeable than autotrophic picoplankton. The contribution of HNA cells was generally low in the upper layers, ranging from 36% in late spring and early summer to ca. 50% in winter and fall. Autotrophs dominated integrated picoplankton biomass in the upper 100 m, with 1.4-fold higher values in summer than in winter (mean 387 and 272 mg C m^–2^, respectively). However, when the whole water column was considered, the biomass of heterotrophic prokaryotes exceeded that of autotrophic picoplankton with an average of 411 mg C m^–2^. Despite being located in tropical waters, our results show that the picoplankton community seasonal differences in the central Red Sea are not fundamentally different from higher latitude regions.

## Introduction

Picoplankton comprises both autotrophic and heterotrophic unicellular organisms in the size range of 0.2 to 2 µm. Picocyanobacteria of the genera *Prochlorococcus* (typically 0.6–0.8 µm in diameter) and *Synechococcus* (ca. 1 µm) usually dominate numerically autotrophic picoplankton, which also includes a high diversity of picoeukaryotes larger than 1 µm (Campbell et al., 1997; Giovannoni & Vergin, 2012). *Prochlorococcus* is usually more abundant than *Synechococcus* in highly stratified and low-nutrient surface waters (DuRand, Olson & Chisholm, 2001; Giovannoni & Vergin, 2012; Olson et al., 1990; Zubkov et al., 2000; Zwirglmaier et al., 2007). Picoeukaryotes are less abundant than picocyanobacteria (Kirkham et al., 2013), especially in the tropical and subtropical oceans (Kirkham et al., 2013; Morán, Fernández & Pérez, 2004). Heterotrophic picoplankton are mostly prokaryotes, overwhelmingly dominated by bacteria over archaea in the upper layers since the abundance of the latter only increases significantly at depth (Karner, DeLong & Karl, 2001). Two flow cytometric populations of heterotrophic prokaryotes are typically detected after staining with DNA-binding dyes: high (HNA) and low (LNA) nucleic acid content cells (Gasol et al., 1999; Li, Jellett & Dickie, 1995; Nishimura, Kim & Nagata, 2005; Sherr, Sherr & Longnecker, 2006). The HNA group typically dominates in eutrophic and mesotrophic conditions characterizing the colder, nutrient-rich months while LNA tends to dominate in stratified oligotrophic environments (Calvo-Díaz & Morán, 2006; Morán et al., 2007; Zubkov, Allen & Fuchs, 2004).

Seasonal changes in the abundance of autotrophic picoplankton groups are well known in temperate (Calvo-Díaz & Morán, 2006; Li, 1998; Morán, 2007) and polar waters (Iversen & Seuthe, 2011; Rivkin, 1991) while they are less known in lower latitude waters, with the exception of two long-term sites: the Bermuda Atlantic Time Series (BATS) in the western Sargasso Sea (DuRand, Olson & Chisholm, 2001; Malmstrom et al., 2010) and the Hawaii Ocean Time-series (HOT) in the North Pacific subtropical gyre (Campbell et al., 1997; Malmstrom et al., 2010). In contrast to autotrophs, the seasonality of heterotrophic bacteria in subtropical and tropical waters is thought to be less pronounced than in temperate regions (Bunse & Pinhassi, 2017).

The Red Sea is an oligotrophic marine basin with very high temperatures (up to 35 ° C at the surface in summer, Calbet et al., 2015; Chaidez et al., 2017; Rasul, Stewart & Nawab, 2015) and salinities (ca. 40, Tesfamichael & Pauly, 2016). The effect of these quasi-extreme conditions on the seasonality of picoplankton communities has received far less attention than other oligotrophic waters. Understanding the temporal changes of picoplankton abundance and their response to environmental drivers are essential to define the lower trophic levels of Red Sea pelagic food webs. Regarding autotrophs, we have a good understanding of their seasonal variability (Al-Najjar et al., 2007; Lindell & Post, 1995; Post et al., 2011) and their trophic relationships with other components of the microbial food web in the northern reaches, especially in the Gulf of Aqaba (Berninger & Wickham, 2005; Sommer, 2000; Sommer et al., 2002). For heterotrophic prokaryotes, although our knowledge about their diversity is increasing (Ngugi et al., 2012; Pearman et al., 2017; Thompson et al., 2017), only a few studies have investigated their vertical distribution in Red Sea waters (Calbet et al., 2015; Qian et al., 2011). A recent report using data collected from the same site as this study has shown that the abundance of heterotrophic bacteria can change temporally up to 3-fold within the same depth in the upper epipelagic (Calleja, Al-Otaibi & Morán, 2019; García et al., 2018). Other studies conducted at that site have shown that LNA bacteria dominated in the epipelagic layer, while HNA cells were more abundant in the mesopelagic layer, indicating that each group seems to prefer different environmental conditions (Calleja, Al-Otaibi & Morán, 2019; Calleja et al., 2018). The unexpectedly low standing stocks of heterotrophic bacteria in a nearby shallow embayment have been explained by strong top-down control exerted by protistan grazers and viruses (Sabbagh et al. submitted; Silva et al., 2019).

Here, we conducted a detailed investigation of both the temporal and vertical variability of autotrophic and heterotrophic picoplankton, as assessed by flow cytometry, by periodic sampling over two years (2015–2017) at a mesopelagic station (ca. 700 m depth) in the central Red Sea, Saudi Arabia. Given the tropical characteristics of the site, we hypothesize that the seasonal variability of picoplankton in epipelagic waters would be lower than that found at higher latitudes, and the marked stratification found between 100 and 200 m should result in strong vertical gradients in abundance, size and ultimately biomass.

## Materials & Methods

### Sample collection and environmental properties

Periodic samplings were conducted from January 2015 to May 2017 on board of RV Thuwal at a mesopelagic station (ca. 700 m depth) located in the central Red Sea, 6 km off the coast of King Abdullah Economic City (KAEC) in Saudi Arabia. Sailing permission were approved by Saudi Coast Guard. We performed 15 vertical profiles evenly distributed along the four seasons (only winter had 3 samples rather than 4, Table 1). Samples were taken at regular depths from the surface to the bottom: 5, 20, 40–80 targeting the deep chlorophyll maximum (DCM), 100, 200, 300, 400, 550, 600 and 700 m. Temperature, salinity, fluorescence and photosynthetically active radiation (PAR) data were acquired with SeaBird SB9 Plus or IDRONAUT 305 CTDs. PAR was available for only 7 sampling times. The depth of the photic layer was determined by the vertical light attenuation coefficient (Kd) as the depth receiving 1% of surface irradiance (Calvo-Díaz & Morán, 2006). Stratification index (SI) was calculated as the density at 100 m minus that at the surface (Calvo-Díaz & Morán, 2006). The upper mixed layer (UML) depth was determined as the first depth in which the difference in density with the shallower 5 m was ≥ 0.05 kg m^−3^ (Calvo-Díaz & Morán, 2006).

**Table 1:** Seasonal distribution and date of the 15 individual samplings at the study site, with the corresponding day of the year for the assessment of seasonal patterns.

**Season**	**Sampling date** **(dd/mm/yyyy)**	**Day of year**
Winter	19∕01∕2015	18
	02∕02∕2016	32
	25∕02∕2017	55
Spring	24∕03∕2015	82
	06∕03∕2016	65
	24∕04∕2017	113
	5∕22∕2017	141
Summer	01∕07∕2015	181
	25∕08∕2015	236
	05∕09∕2015	247
	21∕06∕2016	172
Fall	26∕10∕2015	298
	11∕11∕2015	314
	09∕12∕2015	342
	10∕26∕2016	299

Water samples were taken from Niskin bottles in a rosette sampler with an attached CTD probe (Fig. S1). In 2015 total chlorophyll *a* concentration (Chl *a*) was obtained after filtering 500 to 2,000 ml of the sample through Whatman GF/F filters (25 mm diameter). After checking for the minimum volume yielding reliable results, in 2016 and 2017 we performed sequential filtration of 200 ml samples through filters of 20, 2 and 0.2 µm of pore-size (IsoporeTM Membrane Filters, RTTP, 47 mm diameter), so that Chl *a* was the sum of the corresponding size-fractions: micro- (above 20 µm), nano- (between 2 and 20) and picophytoplankton (between 0.2 and 2 µm). Filters were frozen at −80 °C until analysis in the laboratory. Pigments were extracted in 90% acetone for 24 h in the dark at 4 °C and chlorophyll *a* fluorescence was measured with a Trilogy fluorometer (Turner) calibrated with pure extracts.

Samples for dissolved inorganic nitrogen (DIN = NO^3−^ + NO^2−^), dissolved inorganic phosphorus (DIP = PO_4_^3−^), dissolved organic carbon (DOC) and total dissolved nitrogen (TDN) were filtered through pre-combusted GF/F filters and analyzed as previously reported by Calleja et al. (2018). The nutricline depth was defined as the depth where nitrate concentration first reached 1 µmol L^−1^ (Calvo-Díaz & Morán, 2006).

### Analysis of picoplankton by flow cytometry

Picoplankton samples (1.8 mL) were preserved with 1% paraformaldehyde + 0.05% glutaraldehyde final concentration and placed in the dark for approximately 10 min, then frozen in liquid nitrogen and stored at −80 °C once in the laboratory. After thawing, samples were analyzed with a FACSCanto II flow cytometer (BD-Biosciences). Molecular Probes fluorescent latex beads of 1 µm were used as an internal standard for size and fluorescence measurements. We analyzed aliquots of 0.6 mL for autotrophs and 0.4 mL for heterotrophs, at high (mean 117.9 µL min^−1^) and low (17.9 µL min^−1^) flow rates, respectively, until acquiring 10,000 events. Before analysis, heterotrophic bacteria were stained with 2.5 µmol L^−1^ of the DNA fluorochrome SYBR Green II (Gasol & Morán, 2015). All cytograms were analyzed with FCSExpress 5 software. Autotrophic prokaryotic cells were classified as cyanobacteria (*Synechococcus* and *Prochlorococcus*) and picoeukaryotes according to their orange (PE, 433 nm) and red (PerCP-Cy5-5, 498 nm) fluorescence and light scatter at 90° or side scatter (SSC) signals. Two groups of heterotrophic prokaryotes were distinguished based on their relative green fluorescence (FITC, 360 nm) signal: low and high nucleic acid content (LNA and HNA, respectively). Cell size was determined by an empirical calibration between relative SSC and cell diameter according to Calvo-Díaz & Morán (2006). Spherical shape was assumed for all groups for estimating biovolume, which was transformed into individual biomass by using the biovolume-to-carbon conversion factor of 237 fg C µm^−3^ for autotrophs (Worden, Nolan & Palenik, 2004) and the equation biomass = 108. 8 × (biovolume)^0.898^ for heterotrophs (Gundersen et al., 2002). The biomass of each picoplanktonic group was finally obtained by multiplying the individual biomass estimate by the corresponding abundance.

### Statistical analyses

Picoplankton abundance, biovolume and biomass data were log10-transformed to attain normality and assess their relationship with environmental variables by Spearman’s rank correlation coefficient. One-way ANOVAs and post hoc Tukey’s pairwise comparisons were used to determine significant variations between seasons (*P* < 0.05) with OriginPro software. A non-metric multidimensional scaling (NMDS), a distance-based ordination technique, was performed on the Bray-Curtis dissimilarity distances together with pairwise PERMANOVAs in order to summarize the seasonal and vertical changes in the abundance of the different picoplankton groups and their relation with environmental variables in the upper epipelagic zone. Four groups of samples were considered according to depth: surface, above DCM, DCM depth, below DCM and 100 m. NMDS stress values, a measure of goodness-of-fit, can be used to evaluate the proper choice of dimensions. Low values (0.05–0.1) provide a good fit in reduced dimensions while values >0.3 indicate that the ordination is arbitrary and potentially uninterpretable (Ramette, 2007; Zhu & Yu, 2009). The NMDS analysis was done in R (http://www.r-project.org) and we used the “envfit” function in order to estimate the correlations between the environmental variables and the NMDS axis scores.

## Results

### Vertical and seasonal variability in hydrographic conditions

**Figure 1:**
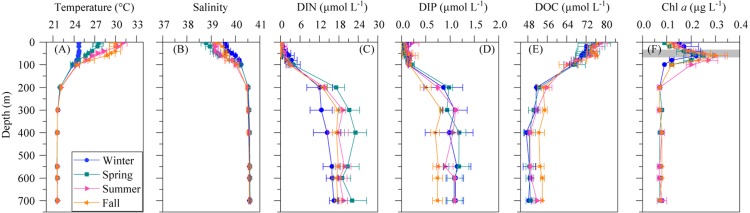
Mean seasonal vertical profiles of environmental variables. (A) temperature, (B) salinity, (C) dissolved inorganic nitrogen (nitrate + nitrite, DIN), (D) dissolved inorganic phosphorus (DIP), (E) dissolved organic carbon (DOC), and (F) chlorophyll *a* (Chl *a*) concentrations in winter, spring, summer and fall at the study station. Error bars show the standard error of the mean. The shaded area in (F) indicates the overall range of the deep chlorophyll maximum (DCM). See the text for details.

The mean vertical profiles of selected environmental variables for the four seasons are shown in Fig. 1. As expected, significant differences (ANOVA: *F* = 14.4, *p* = 0.0004, *n* = 15) in mean surface temperature were found, with summer values 6.3 °C higher than in winter (Table 2). The temperature remained constant year-round from 200 m down to the bottom at 21.7 ± 0.02 °C SE (Fig. 1A). Surface salinity displayed slight seasonal variations from 38.8 ± 0.2 in spring to 39.6 ± 0.1 in winter (Table 2), but there was no seasonal difference below 200 m (40.6 ± 0.0). Differences in SI were not significant despite some seasonality (Table 2), but the UML was significantly shallower in summer than in the other seasons (Table 2, ANOVA: *F* = 18.3, *p* = 0.0002, *n* = 14). The euphotic layer depth varied from 63 to 89 m, with similar mean values across seasons (Table 2).

**Table 2:** Average seasonal values of environmental properties at the surface of the study site and characteristic depths (mean ± SE). Sea surface temperature (SST), salinity, dissolved inorganic nitrogen (nitrate + nitrite, DIN), dissolved inorganic phosphorus (phosphate, DIP), dissolved organic carbon (DOC), total chlorophyll *a* concentration (Chl *a*), stratification index (SI) and depths of the upper mixed layer (UML), the euphotic zone (Zph), the deep chlorophyll maximum (DCM) and the nutricline (NC). Stars and superscript letters indicate significant differences between seasons (ANOVA and Tukey post hoc test; *, *p* = 0.05; ** *p* = 0.01; *** *p* = 0.001.

	**Winter**	**Spring**	**Summer**	**Fall**
SST *** (°C)	24.7 ± 0.1 ^a^ *n* = 3	27.5 ± 0.7 ^a,c^ *n* = 4	30.9 ± 0.7 ^b,c^ *n* = 4	29.9 ± 0.8 ^c^ *n* = 4
**Salinity**	39.6 ± 0.1 *n* = 3	38.8 ± 0.2 *n* = 4	39.2 ± 0.2 *n* = 4	39.2 ± 0.2 *n* = 4
**DIN** **(µmol l**^−1^**)**	0.1 ± 0.04 *n* = 2	0.2 ± 0.1 *n* = 4	0.3 ± 0.2 *n* = 2	0.2 ± 0.1 *n* = 3
**DIP** **(µmol l**^−1^**)**	0.1 ± 0.1 *n* = 2	0.04 ± 0.01 *n* = 4	0.2 ± 0.1 *n* = 2	0.1 ± 0.04 *n* = 3
**DOC** **(µmol l**^−1^**)**	75.8 ± 1.4 *n* = 2	75.4 ± 6.7 *n* = 4	77.7 ± 2.9 *n* = 4	75.3 ± 1.7 *n* = 3
**Chl***a* **(µg l**^−1^**)**	0.15 ± 0.05 *n* = 2	0.09 ± 0.003 *n* = 4	0.13 ± 0.02 *n* = 4	0.12 ± 0.02 *n* = 4
**SI**	1.03 ± 0.14 *n* = 2	2.68 ± 0.52 *n* = 4	2.74 ± 0.27 *n* = 4	2.39 ± 0.26 *n* = 4
UML *** (m)	60 ± 7 ^a^ *n* = 2	43 ± 2 ^a^ *n* = 4	19 ± 3 ^b^ *n* = 4	48 ± 5 ^a^ *n* = 4
**Zph (m)**	85 ± 1 *n* = 2	85 ± 2 *n* = 3	76 ± 4 *n* = 4	72 ± 4 *n* = 4
**DCM (m)**	55 ± 1 *n* = 4	62 ± 8 *n* = 4	63 ± 8 *n* = 4	44 ± 12 *n* = 4
**NC (m)**	63 ± 7 *n* = 2	74 ± 8 *n* = 4	47 ± 35 *n* = 2	75 ± 5 *n* = 3

Dissolved inorganic nitrogen (DIN = nitrate + nitrite) presented uniformly low concentrations at the surface (0.17 ± 0.11 µmol L^−1^) but reached 20.5 ± 1.6 µmol L^−1^ at depths higher than 200 m (Fig. 1C), with an average nutricline depth of 67 ± 6 m (Table 2). Dissolved inorganic phosphate (DIP) followed the same pattern as DIN, with low seasonal mean values at the surface (0.10 ± 0.04 µmol L^−1^) and increasing with depth to a seasonal mean maximum of 1.17 ± 0.1 µmol L^−1^at around 600 m depth (Fig. 1D). The concentration of dissolved organic carbon (DOC, Fig. 1E) declined with depth from a mean 76.1 ± 7.5 µmol L^−1^ at the surface to 52.2 ± 5.8 µmol L^−1^ below 200 m.

### Total and size-fractionated chlorophyll *a* concentration

**Figure 2:**
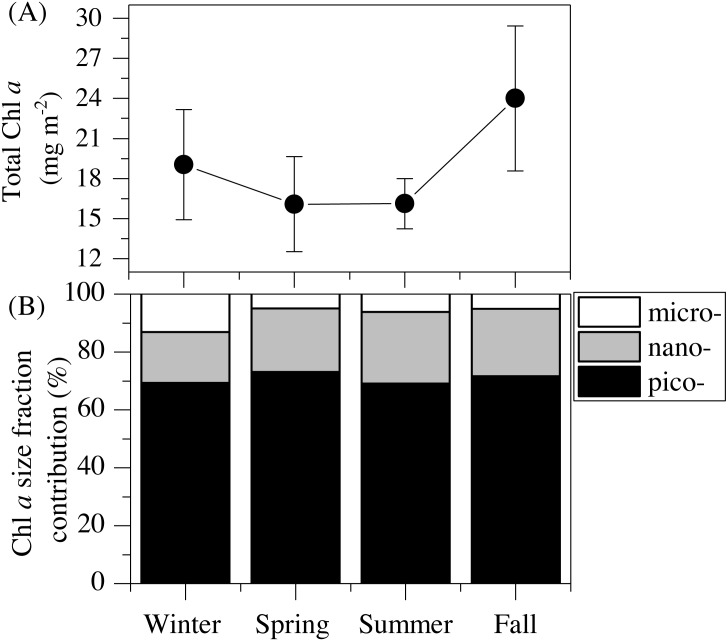
Mean seasonal values of total and size-fractionated chlorophyll *a* concentration. (A) total integrated chlorophyll *a* concentration and (B) mean contributions of the three size-fractions in the upper 100 m of the study station. pico- : picoplankton, nano- : nanoplankton and micro- : microplankton.

The vertical distribution of total chlorophyll *a* (Chl *a*) concentration (Fig. 1F) showed a consistent and clear deep chlorophyll maximum (DCM) located at an average depth of 56 ± 4 m (Table 2). Surface seasonal mean values ranged from 0.09 to 0.15 µg L^−1^ (Fig. 1F). Mean integrated Chl *a* values for the upper 100 m increased from 16.1 mg m^−2^in spring and summer (±2.06 and 1.09, respectively) to 19.0 ± 2.9 mg m^−2^ in winter and 24.0 ± 2.7 mg m^−2^ in fall (Fig. 2A). The picoplankton size fraction contributed, on average, 70.8 ± 1.0% to total integrated values, with nanoplankton and microplankton making up 21.9 ± 1.5% and 7.3 ± 1.9%, respectively, with no significant differences in the relative contributions of the three size classes (Fig. 2B).

### Vertical distribution of picoplankton abundance and cellular characteristics

**Figure 3:**
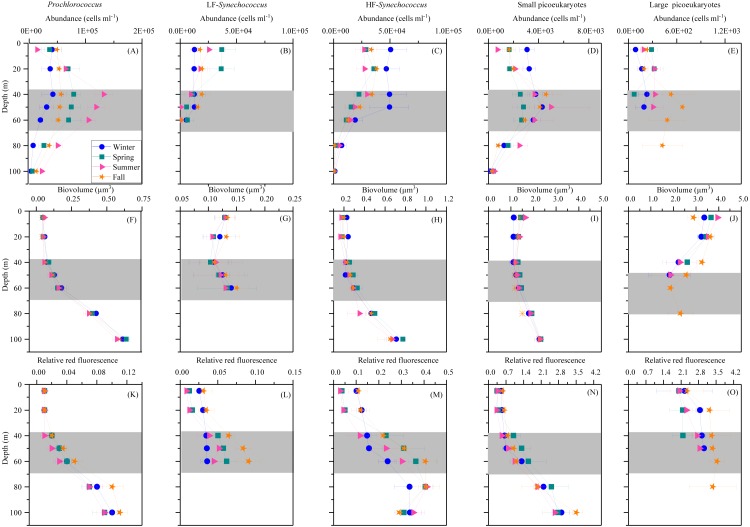
Vertical distribution of autotrophic picoplankton mean seasonal abundance and cellular characteristics. (A–E) abundance, (F–J) biovolume,(K–O) relative red fluorescence of *Prochlorococcus*, low (LF-Syn) and high (HF-Syn) phycoerythrin fluorescence populations of *Synechococcus* and small and large picoeukaryotes in winter, spring, summer and fall at the study station. Error bars show the standard error of the mean. The shaded area indicates the overall range of the DCM.

*Prochlorococcus*, *Synechococcus* and picoeukaryotes were mostly restricted to the upper 100 m, with none of the groups detected in significant numbers at or below 150 m depth. Figure 3 shows the average vertical distribution of picophytoplankton abundance, cell size and relative red fluorescence (as a proxy of Chl *a* content) for each season. *Prochlorococcus* abundance was generally low at the surface (1.11–5.81 × 10^4^ cells mL^−1^) and peaked at the DCM (1.32 ± 0.16 × 10^5^ cells mL^−1^ in summer) (Fig. 3A). The two groups of *Synechococcus* discriminated by low (LF-Syn) and high (HF-Syn) phycoerythrin fluorescence were consistently less abundant than *Prochlorococcus*. LF-Syn and HF-Syn tended to show higher numbers in the surface layers, with averages of 2.29 ± 0.53 × 10^4^ and 3.47 ± 0.54 × 10^4^ cells mL^−1^, respectively (Figs. 3B and 3C). HF-Syn reached deeper than LF-Syn with the latter virtually absent at 80 m (Figs. 3B and 3C). Two groups of picoeukaryotes according to size were consistently distinguished, hereafter referred to as Small (Speuk) and large (Lpeuk). Speuk vertical distribution was similar to that of *Prochlorococcus* (Fig. 3D), while Lpeuk usually disappeared deeper than 40–60 m except in fall, where the highest values were found in the DCM (Figs. 3D and 3E). Coincident with declining abundances, the biovolume of all groups increased steadily with depth from 40 m downwards except for Lpeuk (Figs. 3F–3J). Similar to biovolume, the relative red fluorescence increased consistently with depth, with less marked patterns for LF-Syn and Lpeuk due to their shallower distribution (Figs. 3K–3O).

**Figure 4:**
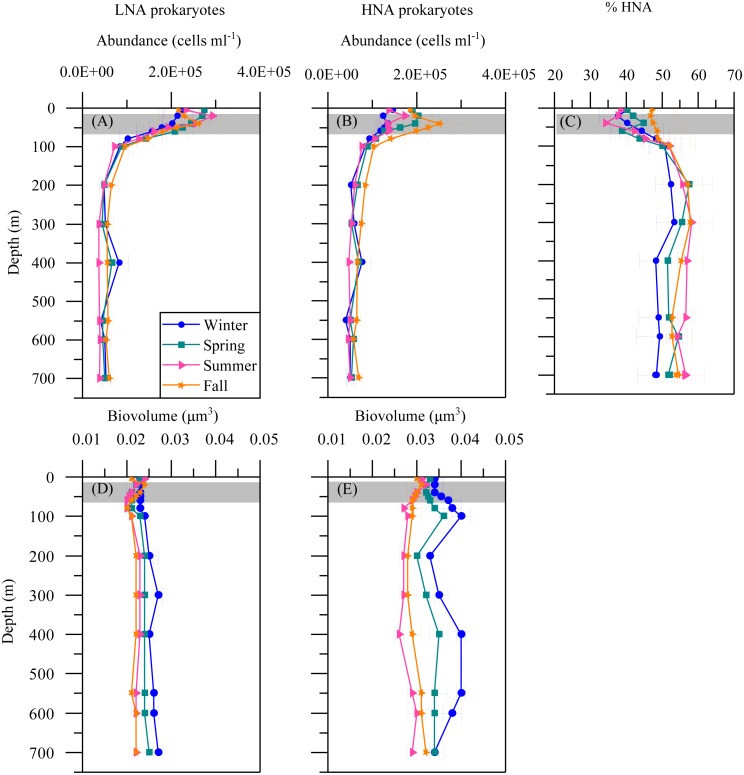
Vertical distribution of heterotrophic prokaryotes mean seasonal abundance and biovolume. Abundance of LNA (A) and (B) HNA cells, (C) contribution of HNA cells to total numbers (%HNA) and biovolume of LNA (D) and HNA (E) cells in winter, spring, summer and fall at the study station. Error bars show the standard error of the mean. The shaded area indicates the overall range of the DCM.

The mean seasonal distribution of heterotrophic prokaryotes abundance and cell size with depth is shown in Fig. 4. The abundances of both LNA and HNA cells were highest in the upper 100 m (maxima of 2. 92 × 10^5^ and 2. 51 × 10^5^ cells mL ^−1^a t 20 and 40 m, respectively) but remained relatively stable for the entire mesopelagic layer (Figs. 4A and 4B). LNA were more abundant than HNA cells in the upper epipelagic, resulting in a contribution of HNA cells to total abundance (%HNA) that ranged from 38.3 to 47.1% at the surface. Values increased to 52.3 –57.4% at 200 m and remained pretty homogeneous down to the sea floor (Fig. 4C). The biovolume of HNA cells was consistently larger than that of LNA cells throughout the water column. Differences were observed between seasons, with maxima in winter for both groups and minima in summer for HNA and in fall for LNA cells (Figs. 4D and 4E).

### Seasonal variation of picoplankton abundance and cellular characteristics

**Figure 5:**
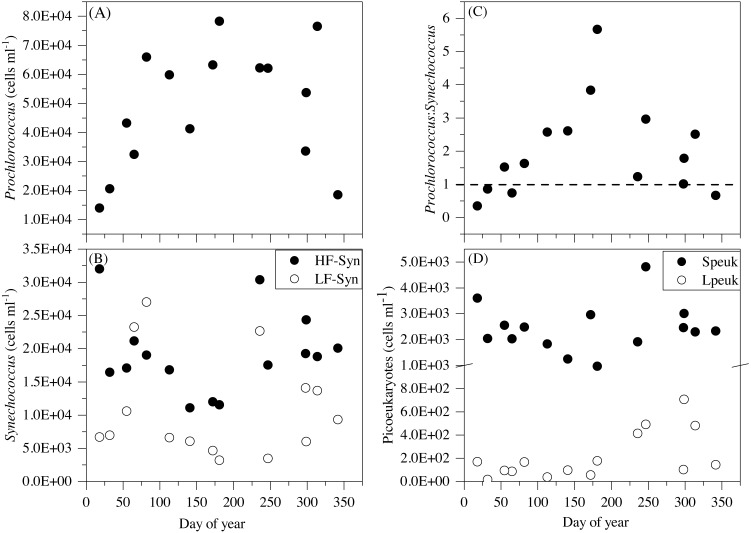
Temporal variability of autotrophic picoplankton abundances averaged for the upper 100 m. (A) *Prochlorococcus*, (B) high (HF-Syn) and low fluorescence (LF-Syn) *Synechococcus* and (D) small (Speuk) and large (Lpeuk) picoeukaryotes. Also shown in (C) the ratio between *Prochlorococcus* and *Synechococcus* cell abundances.

Although some differences between seasons were already apparent in the vertical distributions (Figs. 3 and 4), depth-weighted averages from the surface to 100 m were calculated to better capture the seasonal changes. *Prochlorococcus* abundance displayed a clear seasonal pattern, with minimum values in winter (1.4 × 10^4^ cells mL^−1^, January 2015) and maximum values in summer (7.8 × 10^4^ cells mL^−1^, July 2015) (Fig. 5A). The two groups of *Synechococcus* shared similar dynamics, with maximum values in spring for LF-Syn (2.7 × 10^4^ cells mL^−1^, March 2015) and winter for HF-Syn (3.2 × 10^4^ cells mL^−1^, January 2015) while the lowest values were observed between late spring and early summer for both groups (Fig. 5B). Consequently, the ratio between *Prochlorococcus* and *Synechococcus* abundance, which was higher than 1 for most of the year, was occasionally lower in winter and at the end of fall (Fig. 5C). Speuk abundance presented low values in summer (9.9 × 10^2^ cells mL^−1^, July 2015) and higher in winter (3.60 × 10^3^ cells mL^−1^, January 2015) while Lpeuk, generally less abundant, peaked in fall (7.1 × 10^2^ cells mL^−1^, October 2016) (Fig. 5D). On an annual basis, *Prochlorococcus* contributed 57.6 ± 4.2% to total picophytoplankton cell numbers, followed by *Synechococcus* (38.9 ± 3.9%) with picoeukaryotes 21- to 79-fold lower abundances than cyanobacteria. Depth-weighted biovolumes (0.14–0.25 µm^3^ *Prochlorococcus*, 0.08–0.19 µm^3^ LF-Syn, 0.27–0.42 µm^3^ HF-Syn, 1.09–1.66 µm^3^ Speuk, 2.50–3.62 µm^3^ Lpeuk) did not show any clear seasonal pattern with slightly increased values of Speuk in early summer and Lpeuk in fall (Figs. S2A–S2C). The seasonality of 0–100 m mean relative red fluorescence as a proxy for Chl *a* content followed the expected summer minimum only for *Prochlorococcus* and LF-Syn (Figs. S2D–S2F). Differences in biovolume affected little the changes mentioned above in abundance when calculating the biomass of the different picophytoplankton groups. Integrated autotrophic picoplankton biomass for the upper 100 m showed higher values in summer (387.4 mg C m^−2^) with a significant contribution of *Prochlorococcus* (46.6 ± 6%) (ANOVA: *F* = 4.2, *p* = 0.03, *n* = 15) except in winter when *Synechococcus* contributed 49.5% with a high contribution of HF-Syn (37.02%).

**Figure 6:**
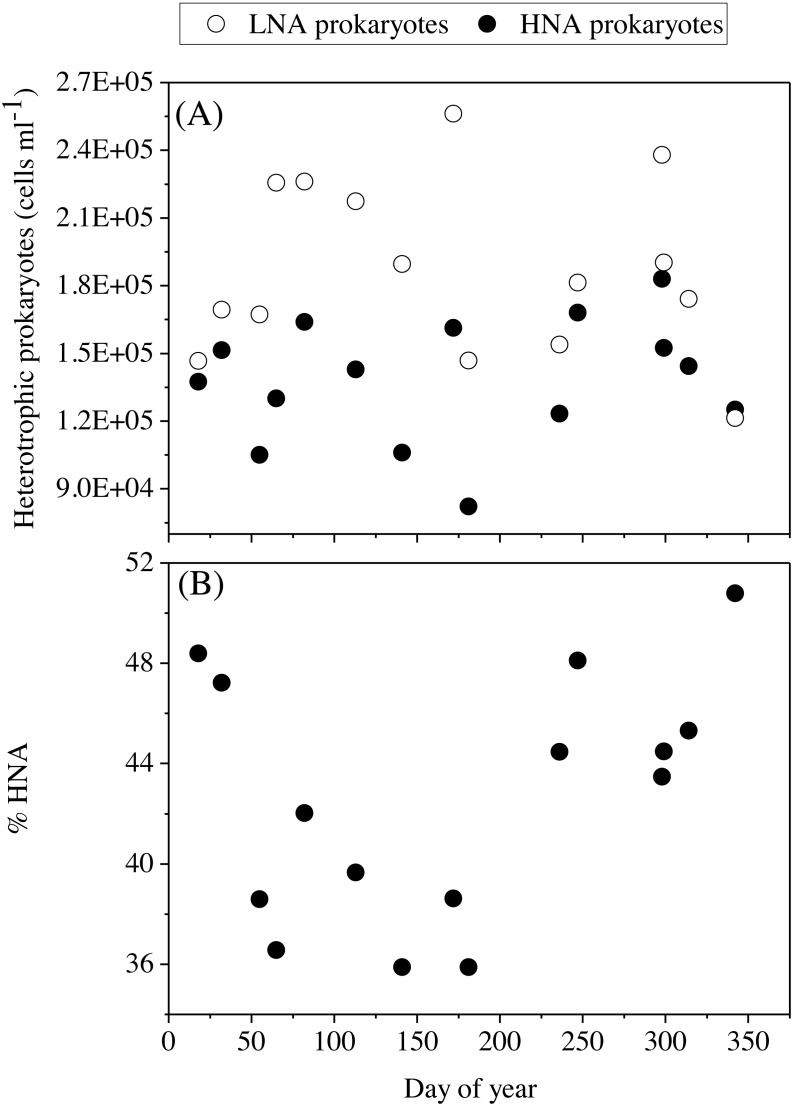
Temporal variability of heterotrophic prokaryotes abundances averaged for the upper 100 m. (A) low (LNA) and high (HNA) nucleic acid bacteria and (B) contribution of HNA cells to total numbers (%HNA).

**Figure 7:**
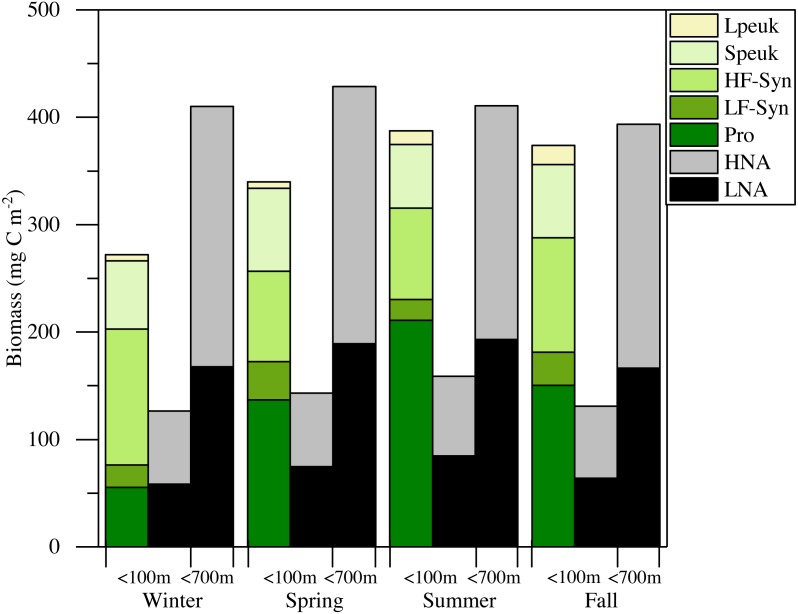
Mean seasonal values of autotrophic and heterotrophic picoplankton integrated biomass in winter, spring, summer and fall at the study station. The green-yellow bar shows**** the integrated biomass for the upper 100 m of autotrophic picoplankton (*Prochlorococcus* (Pro), low (LF-Syn) and high (HF-Syn) phycoerythrin fluorescence populations of *Synechococcus* and small (Speuk) and large (Lpeuk) picoeukaryotes). The first black-gray bar shows the same for heterotrophic prokaryotes (<100 m) while the second black-gray bar shows the values integrated through the entire water column (<700 m).

The mean total abundance of heterotrophic prokaryotes (HNA + LNA prokaryotes) in the upper 100 m ranged from 2.29 to 4.21 × 10^5^ cells mL^−1^, with higher values in spring and fall and lower in summer (Dataset S1). Figure 6A shows the corresponding values for the LNA and HNA groups with respective annual means of 1.87 ± 0.01 × 10^5^ and 1.38 ± 0.07 × 10^5^ cells mL^−1^. Although their abundances failed to show marked seasonal patterns, a clear seasonality in the contribution of HNA bacteria emerged. Upper epipelagic-averaged %HNA values ranged from 35.9% in late spring and early summer to ca. 50% in winter and fall (Fig. 6B). In contrast, the seasonality in biovolume and relative nucleic acid content was not clear for any of the two groups (Figs. S3A and S3B). The integrated biomass of heterotrophic prokaryotes in the upper 100 m ranged from 86.9 to 257.6 mg C m^−2^, with seasonal means shown in Fig. 7. Regarding the contribution of LNA and HNA cells to total heterotrophic prokaryotes biomass in the upper epipelagic, differences were minor with the HNA group prevailing in winter (53.8%) and fall (51.3%) and the LNA groups in summer (53.4%) and spring (52.3%) (Fig. 7). Overall, the biomass of autotrophic picoplankton groups was consistently higher than that of heterotrophic bacteria in the upper epipelagic, with annual averages of 348.1 ± 20.5 and 140.8 ± 10.1 mg C m^−2^, respectively. However, when values were integrated over the entire water column (0–700 m), the mean biomass of heterotrophic bacteria increased to 410.7 ± 27.0 mg C m^−2^, thus exceeding the total biomass of autotrophic picoplankton (Fig. 7). Moreover, when the entire water column was considered HNA cells clearly dominated total biomass regardless of the season.

### Relationships with environmental variables

**Figure 8:**
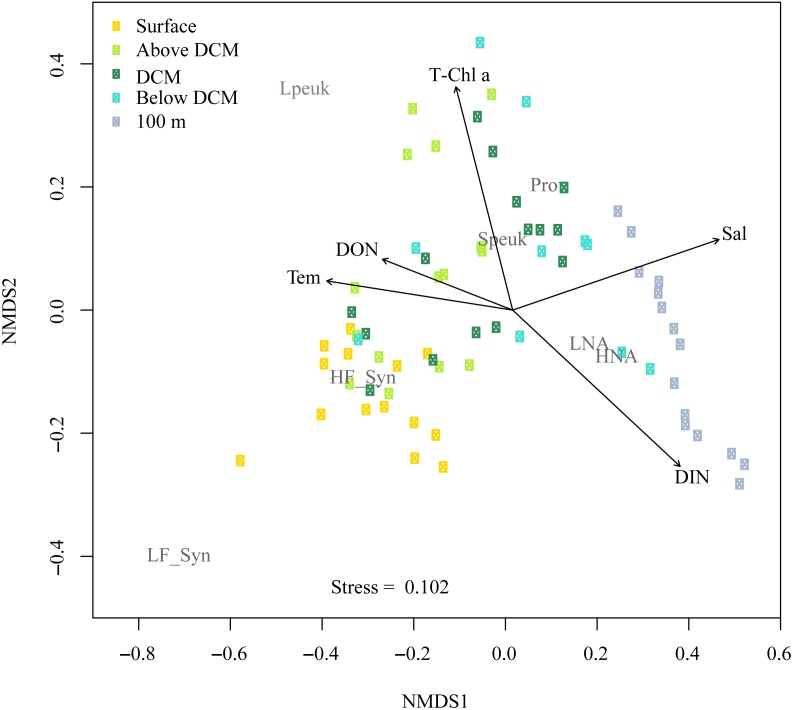
Nonmetric multidimensional scaling (NMDS) analysis of Bray–Curtis distances of the abundances of autotrophic and heterotrophic picoplankton and significant environmental variables in the upper epipelagic zone. All samples were arranged by five depth categories (surface, above DCM, DCM, below DCM and 100 m) and coupled to significant (*p* < 0.05) environmental variables: temperature (Tem), salinity (Sal), total chlorophyll *a* (T-Chl *a*), dissolved inorganic nitrogen (DIN) and dissolved organic nitrogen (DON) concentration. The centroids of the different populations (labeled in gray) indicate where each population was more abundant, and the vectors indicate the direction and strength of the environmental parameters. Group abbreviations as in Fig. 7 and in the main text.

Figure 8 shows the NMDS performed on the Bray-Curtis distances of the abundances for autotrophic and heterotrophic picoplankton populations at different depths in the upper epipelagic (Fig. 8). The low stress value (0.1) indicated a reliable distribution of the samples in two dimensions. All environmental variables were initially considered in the NMDS analysis, but some of them (e.g., DIP, DOC, UML, etc.) were removed since they did not show significant effects on the distribution of the samples. The correlation of the NMDS scores (position of the samples) with the environmental variables (represented by the arrows) indicated significant effects of temperature (*r* = 0.66, *p* = 0.002), DON (*r* = 0.48, *p* = 0.034, Chl *a* (*r* = 0.62, *p* = 0.003), DIN (*r* = 0.72, *p* = 0.001) and salinity (*r* = 0.75, *p* = 0.001). The NMDS plot also showed different habitat segregation of the picoplanktonic groups, with an overall significant effect of the depth layer (PERMANOVA: r^2^ = 0.53, *p* < 0.01). The most abundant group in the surface was *Synechococcus* (mostly the HF_Syn), where temperature and DON were highest. In contrast, *Prochlorococcus* and picoeukaryotes (mainly Speuk) were more abundant around the depth of the DCM, actually contributing to the increase in Chl *a*. HNA and LNA had a higher weight at 100 m, primarily because of the decrease in autotrophic picoplankton groups, where DIN and salinity values started to increase with depth (Fig. 8). There was no significant clustering of samples according to the different seasons (PERMANOVA: *r*^2^ = 0.04, *p* = 0.33), indicating that the effect of depth layer was stronger than season (Fig. S4).

## Discussion

The Red Sea represents a unique environment to investigate how picoplankton, the dominant planktonic size class at low latitudes (Buck, Chavez & Campbell, 1996; Malmstrom et al., 2010; Olson et al., 1990), respond to some of the highest natural temperatures and salinities that can be found in the ocean. We present here a comprehensive flow cytometric assessment of autotrophic and heterotrophic groups at both the vertical and seasonal scales at a 700-m deep station in the central Red Sea. Surface waters showed persistently high stratification, limiting the availability of DIN and DIP, which resulted in low phytoplankton biomass for most of the year (Figs. 1F and 2) and a clear dominance of small cells consistent with previous work (Bock et al., 2018; Van den Engh et al., 2017; Wei et al., 2019). Accordingly, DOC concentrations did not exceed 95 µmol L^−1^. More information on the hydrological features and DOC dynamics of the study site can be found in Calleja, Al-Otaibi & Morán (2019). In the nearby shallow waters of KAUST Harbor, although conditions were still oligotrophic year-round, higher concentrations of DIN and DOC were occasionally observed (Silva et al., 2019).

### Vertical distribution of picoplankton

In this study, although cyanobacteria and picoeukaryotes made up most of the picophytoplankton biomass as chlorophyll *a* (Fig. 2B), different depth preferences for each group were found. *Prochlorococcus* and the two size fractions of picoeukaryotes tended to show higher abundances at a depth of the DCM, which ranged from 40 to 76 m, than at the surface where both groups of *Synechococcus* peaked (Figs. 3A–3E). This distribution was further confirmed by the NMDS analysis of all samples (Fig. 8), showing a clear cluster of surface samples (with higher temperatures and DON) dominated by *Synechococcus*, while *Prochlorococcus* and picoeukaryotes dominated the DCM. This vertical segregation of *Prochlorococcus* and *Synechococcus* is well known (Partensky, Hess & Vaulot, 1999; Rabouille, Edwards & Zehr, 2007) and indicates a different adaptation to ambient light conditions. The light-harvesting antenna of *Synechococcus* have phycobilisomes with phycobiliproteins (phycoerythrin and phycocyanin) that confer them a higher ability to stand the high irradiances (including UV wavelengths) found at the surface (Biller et al., 2015). Several studies have suggested that *Prochlorococcus* is more sensitive to sunlight, particularly to UV potentially causing DNA-damage, than *Synechococcus* (Agustí, 2004; Boelen et al., 2000; Boelen et al., 2002). Accordingly, *Prochlorococcus* is better adapted to capture the blue wavelengths that predominate deeper in the water column (Biller et al., 2015), thus giving rise to the observed differences in vertical distribution. However, the maximum depth at which we were able to detect cyanobacteria and picoeukaryotes by flow cytometry was generally 100 m. Molecular analysis is more sensitive than flow cytometry at finding rare populations. A recent study in the Red Sea assessing 16S rRNA gene sequences, more sensitive than flow cytometry, was able to find *Prochlorococcus* below 200 m, though with low numbers (Shibl et al., 2016). In other studies conducted in tropical waters, *Prochlorococcus* and *Synechococcus* were, however, detected by flow cytometry at depths of 150 to 200 m (Bock et al., 2018; Partensky, Hess & Vaulot, 1999; Van den Engh et al., 2017). The flow cytometer used in this study has a high sensitivity to detect small cells (<1 µm) and viruses (Monier et al., 2017). Thus, we believe that the apparent disappearance of picophytoplankton at those depths is rather a reflection of the already low numbers found in shallower depths compared with other studies than a problem with the detection limit (Ribeiro et al., 2016). Bearing in mind that 15 profiles along the annual cycle might still have missed the period of highest concentration, to our knowledge, the maximum abundance observed of *Prochlorococcus* (1.63 × 10^5^ cells ml^−1^) lies among the lowest ever recorded. For instance, the maximum values in the subtropical and tropical oceans found during the Malaspina-2010 expedition were 14.2 × 10^5^ cells ml^−1^ for the Atlantic, 6.35 × 10^5^ cells ml^−1^ for the Indian and 3.27 × 10^5^ cells ml^−1^ for the Pacific (Agustí et al., 2019). The mean abundance of *Synechococcus* at the study site was also lower (1.16 × 10^4^ cells ml^−1^) than in the Indian (2.34 × 10^4^ cells ml^−1^) and Pacific (4.85 × 10^4^ cells ml^−1^) oceans, but higher than in the Atlantic (0.87 × 10^4^ cells ml^−1^). Very low abundances of picoeukaryotes were also consistently found in this study (9.7 × 10^3^ cells ml^−1^) compared to the Atlantic (18.5 × 10^3^ cells ml^−1^), Indian (16.7 × 10^3^ cells ml^−1^) and Pacific (79.3 × 10^3^ cells ml^−1^) oceans (Agustí et al., 2019). As in previous reports, consistent associations between the decrease in abundance and the increase in cell size and relative red fluorescence were observed for all picophytoplankton groups except for large picoeukaryotes (Figs. 3F–3O). This increase should be primarily attributed to the combined effects of depth-varying environmental variables such as inorganic nutrients availability and light (Chen et al., 2011), although shifts in species composition may also play a role (Campbell & Vaulot, 1993). The decrease in irradiance drives the need to synthesize more proteins and pigments to capture the fewer photons reaching the deeper layers (Van den Engh et al., 2017).

Regarding the vertical distribution of heterotrophic prokaryotes, we confirm the findings of two recent studies conducted at the same site as ours, focused on the interactions of bacteria with DOC stocks at the diel (García et al., 2018) and seasonal scales (Calleja, Al-Otaibi & Morán, 2019). As previously reported (Calleja, Al-Otaibi & Morán, 2019; García et al., 2018), LNA bacteria dominated in the epipelagic zone while HNA bacteria prevailed in the mesopelagic zone. The lower relative numbers of HNA cells in the upper 100 m (usually below 51%) could be explained by the presence of protistan grazers with a preference for the larger HNA cells (Gonzalez, Sherr & Sherr, 1990; Lefort & Gasol, 2014). Recent work has shown that the abundances of heterotrophic nanoflagellates were negatively correlated with the sizes of both LNA and HNA cells, suggesting a preference to graze on the larger cells from both groups (Sabbagh et al., submitted). In turn, the dominance of HNA cells in the whole mesopelagic layer suggests either a release from grazing pressure (Lara et al., 2017) or that different taxa belonging to the HNA cluster are better suited to exploit the DOC compounds found at depth (Calleja, Al-Otaibi & Morán, 2019). The mesopelagic zone in this Red Sea site is characterized by a deep scattering layer, located between 400 and 600 m, where vertically migrating fish concentrate during the day (Calleja et al., 2018; Røstad, Kaartvedt & Aksnes, 2016). This layer seems to play an essential role in fast carbon transport and cycling by heterotrophic prokaryotes, as shown in previous studies (Calleja, Al-Otaibi & Morán, 2019; García et al., 2018).

Overall, the vertical distribution of picoplankton was most clearly affected by depth, in turn related to strong gradients in environmental variables (temperature, light, UV, inorganic nutrients, etc.), as clearly observed in the NMDS distribution of samples (Fig. 7) that cluster according to layer much more obviously than to season (Fig. S4). However, seasonal patterns became more evident when considering the depth-averaged or integrated values, as discussed below.

### Seasonal variation of picoplankton

Except at very high latitudes (Cottrell & Kirchman, 2009; Li, 2009; Waleron et al., 2007), cyanobacteria numerically dominate picophytoplankton communities, although the prevailing genus depends on the specific physicochemical properties and trophic structure. The dominance of *Prochlorococcus* has been frequently observed in high temperature, low nutrient and stratified waters, while *Synechococcus* and picoeukaryotes are usually predominant at lower temperatures, higher nutrient concentrations and more mixed waters (Campbell et al., 1997; Malmstrom et al., 2010). It has been hypothesized that the seasonality of picoplankton groups in tropical and subtropical oceans is less pronounced than in temperate or polar regions (Bunse & Pinhassi, 2017). However, although our site can be safely considered as permanently oligotrophic since it is strongly stratified year-round, surface temperature did indeed change between seasons (Table 2). On an annual scale, the longest subtropical time series at BATS displays high abundance of *Prochlorococcus* in summer and fall due to strong stratification and low values in late winter due to deep mixing events, while this pattern is much less visible at HOT (Campbell et al., 1997; DuRand, Olson & Chisholm, 2001; Giovannoni & Vergin, 2012; Malmstrom et al., 2010). A similar seasonal variability has also been reported in the Gulf of Aqaba in the northern Red Sea (Al-Najjar et al., 2007). However, two major differences were observed in this study. In spite of the overall dominance of *Prochlorococcus* especially noticeable in summer, *Synechococcus* unexpectedly outnumbered *Prochlorococcus* in winter and fall in the epipelagic layer (Fig. 5C). The fact that two populations of *Synechococcus* of differing orange fluorescence, LF-Syn and HF-Syn, were consistently found year-round did not result in major divergences in seasonality (Fig. 5B). Altogether, the total abundance of *Synechococcus* at our site peaked in winter the same as at HOT station (Campbell et al., 1997; Malmstrom et al., 2010), while the maximum abundance at BATS was found during the spring bloom when the mixed layer deepened and inorganic nutrients were detectable in surface layers (DuRand, Olson & Chisholm, 2001). Picoeukaryotes have been reported to be more abundant in spring at both sites (Campbell et al., 1997; DuRand, Olson & Chisholm, 2001). In our dataset, although the two size fractions demonstrated different seasonality, picoeukaryotes generally tended to peak either at the beginning (winter-spring) or the end of the year (fall) (Fig. 5D).

With complete seasonal coverage, we confirm the finding that LNA heterotrophic prokaryotes dominate in the upper epipelagic (<100 m) while their HNA counterparts prevail in the mesopelagic zone (≥200 m) (García et al., 2018). Heterotrophic bacteria and archaea have been reported to present higher abundances in summer and decline in fall at BATS (Carlson, Ducklow & Sleeter, 1996) while the peak at HOT occurred in summer-fall (Campbell et al., 1997). In this study, the seasonality of both the LNA and HNA groups, as well as their sum, was less noticeable than autotrophic picoplankton, though low numbers were mostly observed in summer, as already reported by Calleja, Al-Otaibi & Morán (2019). Bottom-up control by phosphorus could partially explain the decrease of heterotrophic prokaryotes in summer in the upper 100 m. Calleja, Al-Otaibi & Morán (2019) reported that epipelagic DIN:DIP ratios (without ammonium) peaked at 19 during summer, while lower values of ca. 11 were observed during the rest of the year, concomitant with DOC accumulation that could be a consequence of nutrient-limited and low standing stocks. Concurrently, in the experimental assessment of specific growth rates in the shallow waters of KAUST Harbor, top-down control by protistan grazers has been demonstrated to play an important role in regulating heterotrophic prokaryotes standing stocks (Silva et al., 2019).

There is little information about primary productivity (Qurban, Wafar & Heinle, 2019) and planktonic metabolism (López-Sandoval et al., 2019) in the Red Sea to allow an assessment of the seasonality of its metabolic balance (i.e., the periods of net autotrophy vs. net heterotrophy, García-Martín et al., 2019a; García-Martín et al., 2019b). We can still compare the respective biomasses of autotrophs and heterotrophs within the smaller size fraction, which collectively support to a large extent the higher trophic levels in oligotrophic environments. The relative importance of heterotrophic prokaryotes biomass to total planktonic biomass has been shown to increase with decreasing trophic state (Azam, 1998; Biddanda, Ogdahl & Cotner, 2001; Del Giorgio, Cole & Cimbleris, 1997; Gasol, Del Giorgio & Duarte, 1997), with an average ratio of 1.85 in the oligotrophic ocean (Buck, Chavez & Campbell, 1996; Cho & Azam, 1990; Gasol, Del Giorgio & Duarte, 1997). Considering only picoplankton, autotrophic cells make a higher contribution to total biomass in meso- to eutrophic areas, while heterotrophic bacteria and archaea typically become more important in tropical and subtropical oligotrophic oceans (Harris, Duarte & Nixon, 2006; Regaudie-de Gioux & Duarte, 2013; Zhang, Jiao & Hong, 2008). If we restrict our analysis to the first 100 m, autotrophic picoplankton biomass consistently exceeded that of heterotrophic prokaryotes biomass (Fig. 7), suggesting that the upper central Red Sea would be a net autotrophic ecosystem over the entire annual cycle (i.e., primary production would exceed community respiration), in agreement with the recent study of López-Sandoval et al. (2019). The major contributor to autotrophic picoplankton biomass was *Prochlorococcus* as in other oligotrophic waters (Wei et al., 2019; Zhang, Jiao & Hong, 2008), except in winter. However, if we extend the comparison between autotrophic and heterotrophic picoplankton biomass to the bottom of the study site, the ecosystem would then tend to net heterotrophic, but this difference was not very marked (Fig. 7). It is noteworthy that KAEC station lies between the metabolically balanced or net heterotrophic in the northern Red Sea and the net autotrophic waters of its southern reaches (López-Sandoval et al., 2019). In any case, further studies are necessary to fully understand the functioning of the central Red Sea pelagic ecosystem by a comprehensive assessment of its matter and energy fluxes.

This flow cytometry-based study is the first detailed temporal account of picoplankton abundance, single-cell characteristics and biomass covering from epi- to mesopelagic waters ever conducted in the central Red Sea. Future studies of the variations occurring at the daily scale will help interpret the seasonal patterns of autotrophic and heterotrophic picoplankton described here.

## Conclusion

This work presents different vertical segregation of the picoplanktonic groups surveyed. *Synechococcus* and LNA heterotrophic prokaryotes tended to occupy shallower layers than *Prochlorococcus*, picoeukaryotes and HNA heterotrophic prokaryotes. Seasonality was clearly depicted by the two genera of cyanobacteria, with *Synechococcus* exceeding *Prochlorococcus* cell numbers in early winter and late fall. Picoeukaryotes also tended to be more abundant in winter and fall, contributing to a seasonal structuring of picophytoplankton in Red Sea waters similar to higher latitude ecosystems. The seasonal patterns of heterotrophic prokaryotes were less noticeable than those of picophytoplankton and we did not find clear evidence of higher biomass of picoplanktonic heterotrophs at the study site year-round. Although the vertical gradients in environmental conditions had a major effect on the distribution of autotrophic and heterotrophic picoplankton, temporal changes over the year emerged as an important feature to be considered in future studies of the Red Sea pelagic ecosystem.

##  Supplemental Information

10.7717/peerj.8612/supp-1 Figure S1 Cartoon showing the sampling scheme for field and lab work “created with BioRender.com”

10.7717/peerj.8612/supp-2 Figure S2 Temporal variability of autotrophic picoplankton biovolume and relative red fluorescence as a proxy of chlorophyll *a* content averaged for the upper 100 m (A–C) biovolume and (D–F) relative red fluorescence of *Prochlorococcus*, high (HF-Syn) and low (LF-Syn) phycoerythrin fluorescence *Synechococcus* and small and large (Lpeuk) picoeukaryotes.

10.7717/peerj.8612/supp-3 Figure S3 Temporal variability of heterotrophic prokaryotes biovolume and relative green fluorescence as a proxy of nucleic acid content averaged for the upper 100 m (A) biovolume and (B) nucleic acid content of low (LNA) and high (HNA) nucleic acid bacteria.

10.7717/peerj.8612/supp-4 Figure S4 Nonmetric multidimensional scaling (NMDS) analysis of Bray-Curtis distances of abundances of autotrophic and heterotrophic picoplankton with indication of the different seasons Autotrophic and heterotrophic picoplankton abbreviations as in Fig. 7 and in the main text.

10.7717/peerj.8612/supp-5 Supplemental Information 1 Vertical distribution in hydrographic conditions, picoplankton abundance and cellular characteristics in the central Red Sea

## Additional Information and Declarations

### Field Study Permissions

The following information was supplied relating to field study approvals (i.e., approving body and any reference numbers): Sailing permission approved by Saudi Coast Guard.

### Data Availability

The following information was supplied regarding data availability: The raw data are available as a Supplemental File.

## References

[ref-1] Agustí S (2004). Viability and niche segregation of *Prochlorococcus* and *Synechococcus* cells across the Central Atlantic Ocean. Aquatic Microbial Ecology.

[ref-2] Agustí S, Lubián LM, Moreno-Ostos E, Estrada M, Duarte CM (2019). Projected changes in photosynthetic picoplankton in a warmer subtropical ocean. Frontiers in Marine Science.

[ref-3] Al-Najjar T, Badran MI, Richter C, Meyerhoefer M, Sommer U (2007). Seasonal dynamics of phytoplankton in the Gulf of Aqaba, Red Sea. Hydrobiologia.

[ref-4] Azam F (1998). Microbial control of oceanic carbon flux: the plot thickens. Science.

[ref-5] Berninger U-G, Wickham SA (2005). Response of the microbial food web to manipulation of nutrients and grazers in the oligotrophic Gulf of Aqaba and northern Red Sea. Marine Biology.

[ref-6] Biddanda B, Ogdahl M, Cotner J (2001). Dominance of bacterial metabolism in oligotrophic relative to eutrophic waters. Limnology and Oceanography.

[ref-7] Biller SJ, Berube PM, Lindell D, Chisholm SW (2015). *Prochlorococcus*: the structure and function of collective diversity. Nature Reviews. Microbiology.

[ref-8] Bock N, Van Wambeke F, Dion M, Duhamel S (2018). Microbial community structure in the western tropical South Pacific. Biogeosciences.

[ref-9] Boelen P, De Boer MK, Kraay GW, Veldhuis MJW, Buma AGJ (2000). UVBR-induced DNA damage in natural marine picoplankton assemblages in the tropical Atlantic Ocean. Marine Ecology Progress Series.

[ref-10] Boelen P, Post AF, Veldhuis MJW, Buma AGJ (2002). Diel patterns of UVBR-induced DNA damage in picoplankton size fractions from the Gulf of Aqaba, Red Sea. Microbial Ecology.

[ref-11] Buck K, Chavez F, Campbell L (1996). Basin-wide distributions of living carbon components and the inverted trophic pyramid of the central gyre of the North Atlantic Ocean, summer 1993. Aquatic Microbial Ecology.

[ref-12] Bunse C, Pinhassi J (2017). Marine bacterioplankton seasonal succession dynamics. Trends in Microbiology.

[ref-13] Calbet A, Agersted MD, Kaartvedt S, Møhl M, Møller EF, Enghoff-Poulsen S, Paulsen ML, Solberg I, Tang KW, Tönnesson K (2015). Heterogeneous distribution of plankton within the mixed layer and its implications for bloom formation in tropical seas. Scientific Reports.

[ref-14] Calleja ML, Al-Otaibi N, Morán XAG (2019). Dissolved organic carbon contribution to oxygen respiration in the central Red Sea. Scientific Reports.

[ref-15] Calleja ML, Ansari MI, Rostad A, Silva L, Kaartvedt S, Irigoien X, Morán XAG (2018). The mesopelagic scattering layer: a hotspot for heterotrophic prokaryotes in the red sea twilight zone. Frontiers in Marine Science.

[ref-16] Calvo-Díaz A, Morán XAG (2006). Seasonal dynamics of picoplankton in shelf waters of the southern Bay of Biscay. Aquatic Microbial Ecology.

[ref-17] Campbell L, Liu H, Nolla HA, Vaulot D (1997). Annual variability of phytoplankton and bacteria in the subtropical North Pacific Ocean at Station ALOHA during the 1991–1994 ENSO event. Deep Sea Research Part I: Oceanographic Research Papers.

[ref-18] Campbell L, Vaulot D (1993). Photosynthetic picoplankton community structure in the subtropical North Pacific Ocean near Hawaii (station ALOHA). Deep Sea Research Part I: Oceanographic Research Papers.

[ref-19] Carlson CA, Ducklow HW, Sleeter TD (1996). Stocks and dynamics of bacterioplankton in the northwestern Sargasso Sea. Deep-Sea Research Part Ii-Topical Studies in Oceanography.

[ref-20] Chaidez V, Dreano D, Morán S, Duarte CM, Hoteit I (2017). Decadal trends in Red Sea maximum surface temperature. Scientific Reports.

[ref-21] Chen B, Wang L, Song S, Huang B, Sun J, Liu H (2011). Comparisons of picophytoplankton abundance, size, and fluorescence between summer and winter in northern South China Sea. Continental Shelf Research.

[ref-22] Cho BC, Azam F (1990). Biogeochemical significance of bacterial biomass in the ocean’s euphotic zone. Marine Ecology Progress Series Oldendorf.

[ref-23] Cottrell MT, Kirchman DL (2009). Photoheterotrophic microbes in the arctic ocean in summer and winter. Applied and Environmental Microbiology.

[ref-24] Del Giorgio PA, Cole JJ, Cimbleris A (1997). Respiration rates in bacteria exceed phytoplankton production in unproductive aquatic systems. Nature.

[ref-25] DuRand MD, Olson RJ, Chisholm SW (2001). Phytoplankton population dynamics at the Bermuda Atlantic Time-series station in the Sargasso Sea. Deep Sea Research Part II: Topical Studies in Oceanography.

[ref-26] García FC, Calleja ML, Al-Otaibi N, Røstad A, Morán XAG (2018). Diel dynamics and coupling of heterotrophic prokaryotes and dissolved organic matter in epipelagic and mesopelagic waters of the central Red Sea. Environmental Microbiology.

[ref-27] García-Martín EE, Daniels CJ, Davidson K, Davis CE, Mahaffey C, Mayers KMJ, McNeill S, Poulton AJ, Purdie DA, Tarran GA, Robinson C (2019a). Seasonal changes in plankton respiration and bacterial metabolism in a temperate shelf sea. Progress in Oceanography.

[ref-28] García-Martín EE, Daniels CJ, Davidson K, Lozano J, Mayers KMJ, McNeill S, Mitchell E, Poulton AJ, Purdie DA, Tarran GA, Whyte C, Robinson C (2019b). Plankton community respiration and bacterial metabolism in a North Atlantic Shelf Sea during spring bloom development (2015). Progress in Oceanography.

[ref-29] Gasol JM, Del Giorgio PA, Duarte CM (1997). Biomass distribution in marine planktonic communities. Limnology and Oceanography.

[ref-30] Gasol JM, Morán XAG, McGenity TJ, Timmis KN, Nogales B (2015). Flow cytometric determination of microbial abundances and its use to obtain indices of community structure and relative activity. Hydrocarbon and lipid microbiology protocols: single-cell and single-molecule methods.

[ref-31] Gasol JM, Zweifel UL, Peters F, Fuhrman JA, Hagström Å (1999). Significance of size and nucleic acid content heterogeneity as measured by flow cytometry in natural planktonic bacteria. Applied and Environmental Microbiology.

[ref-32] Regaudie-de Gioux A, Duarte CM (2013). Global patterns in oceanic planktonic metabolism. Limnology and Oceanography.

[ref-33] Giovannoni SJ, Vergin KL (2012). Seasonality in ocean microbial communities. Science.

[ref-34] Gonzalez JM, Sherr EB, Sherr BF (1990). Size-selective grazing on bacteria by natural assemblages of estuarine flagellates and ciliates. Applied and Environmental Microbiology.

[ref-35] Gundersen K, Heldal M, Norland S, Purdie DA, Knap AH (2002). Elemental C, N, and P cell content of individual bacteria collected at the Bermuda Atlantic Time-series Study (BATS) site. Limnology and Oceanography.

[ref-36] Harris LA, Duarte CM, Nixon SW (2006). Allometric laws and prediction in estuarine and coastal ecology. Estuaries and Coasts.

[ref-37] Iversen KR, Seuthe L (2011). Seasonal microbial processes in a high-latitude fjord (Kongsfjorden, Svalbard): I. Heterotrophic bacteria, picoplankton and nanoflagellates. Polar Biology.

[ref-38] Karner MB, DeLong EF, Karl DM (2001). Archaeal dominance in the mesopelagic zone of the Pacific Ocean. Nature.

[ref-39] Kirkham AR, Lepere C, Jardillier LE, Not F, Bouman H, Mead A, Scanlan DJ (2013). A global perspective on marine photosynthetic picoeukaryote community structure. The ISME Journal.

[ref-40] Lara E, Vaqué D, Sa EL, Boras JA, Gomes A, Borrull E, Díez-Vives C, Teira E, Pernice MC, García FC, Forn I, Castillo YM, Peiro A, Salazar G, Morán XAG, Massana R, Catalá TS, Luna GM, Agustí S, Estrada M, Gasol JM, Duarte CM (2017). Unveiling the role and life strategies of viruses from the surface to the dark ocean. Science Advances.

[ref-41] Lefort T, Gasol JM (2014). Short-time scale coupling of picoplankton community structure and single-cell heterotrophic activity in winter in coastal NW Mediterranean Sea waters. Journal of Plankton Research.

[ref-42] Li WKW (1998). Annual average abundance of heterotrophic bacteria and *Synechococcus* in surface ocean waters. Limnology and Oceanography.

[ref-43] Li WKW (2009). From cytometry to macroecology: a quarter century quest in microbial oceanography. Aquatic Microbial Ecology.

[ref-44] Li W, Jellett J, Dickie P (1995). DNA distributions in planktonic bacteria stained with TOTO or TO-PRO. Limnology and Oceanography.

[ref-45] Lindell D, Post AF (1995). Ultraphytoplankton succession is triggered by deep winter mixing in the Gulf-of-Aqaba (Eilat), Red-Sea. Limnology and Oceanography.

[ref-46] López-Sandoval DC, Delgado-Huertas A, Carrillo-de Albornoz P, Duarte CM, Agustí S (2019). Use of cavity ring-down spectrometry to quantify 13C-primary productivity in oligotrophic waters. Limnology and Oceanography: Methods.

[ref-47] Malmstrom RR, Coe A, Kettler GC, Martiny AC, Frias-Lopez J, Zinser ER, Chisholm SW (2010). Temporal dynamics of *Prochlorococcus* ecotypes in the Atlantic and Pacific oceans. ISME Journal.

[ref-48] Monier A, Chambouvet A, Milner DS, Attah V, Terrado R, Lovejoy C, Moreau H, Santoro AE, Derelle E, Richards TA (2017). Host-derived viral transporter protein for nitrogen uptake in infected marine phytoplankton. Proceedings of the National Academy of Sciences of the United States of America.

[ref-49] Morán XAG (2007). Annual cycle of picophytoplankton photosynthesis and growth rates in a temperate coastal ecosystem: a major contribution to carbon fluxes. Aquatic Microbial Ecology.

[ref-50] Morán XAG, Bode A, Suárez LÁ, Nogueira E (2007). Assessing the relevance of nucleic acid content as an indicator of marine bacterial activity. Aquatic Microbial Ecology.

[ref-51] Morán XAG, Fernández E, Pérez V (2004). Size-fractionated primary production, bacterial production and net community production in subtropical and tropical domains of the oligotrophic NE Atlantic in autumn. Marine Ecology Progress Series.

[ref-52] Ngugi DK, Antunes A, Brune A, Stingl U (2012). Biogeography of pelagic bacterioplankton across an antagonistic temperature-salinity gradient in the Red Sea. Molecular Ecology.

[ref-53] Nishimura Y, Kim C, Nagata T (2005). Vertical and seasonal variations of bacterioplankton subgroups with different nucleic Acid contents: possible regulation by phosphorus. Applied and Environmental Microbiology.

[ref-54] Olson RJ, Chisholm SW, Zettler ER, Altabet MA, Dusenberry JA (1990). Spatial and temporal distributions of prochlorophyte picoplankton in the North Atlantic Ocean. Deep Sea Research Part A Oceanographic Research Papers.

[ref-55] Partensky F, Hess WR, Vaulot D (1999). *Prochlorococcus*, a marine photosynthetic prokaryote of global significance. Microbiology and Molecular Biology Reviews.

[ref-56] Pearman JK, Ellis J, Irigoien X, Sarma YVB, Jones BH, Carvalho S (2017). Microbial planktonic communities in the Red Sea: high levels of spatial and temporal variability shaped by nutrient availability and turbulence. Scientific Reports.

[ref-57] Post AF, Penno S, Zandbank K, Paytan A, Huse S, Welch DMark (2011). Long term seasonal dynamics of *Synechococcus* population structure in the Gulf of Aqaba, Northern Red Sea. Frontiers in Microbiology.

[ref-58] Qian PY, Wang Y, Lee OO, Lau SCK, Yang J, Lafi FF, Al-Suwailem A, Wong TYH (2011). Vertical stratification of microbial communities in the Red Sea revealed by 16S rDNA pyrosequencing (vol 5, pg 507, 2010). ISME Journal.

[ref-59] Qurban MAB, Wafar M, Heinle M, Rasul NMA, Stewart ICF (2019). Phytoplankton and primary production in the Red Sea. Oceanographic and biological aspects of the Red Sea.

[ref-60] Rabouille S, Edwards CA, Zehr JP (2007). Modelling the vertical distribution of *Prochlorococcus* and *Synechococcus* in the North Pacific Subtropical Ocean. Environmental Microbiology.

[ref-61] Ramette A (2007). Multivariate analyses in microbial ecology. Fems Microbiology Ecology.

[ref-62] Rasul NM, Stewart IC, Nawab ZA (2015). Introduction to the Red Sea: its origin, structure, and environment.

[ref-63] Ribeiro CGE, Marie D, Dos Santos AL, Brandini FP, Vaulot D (2016). Estimating microbial populations by flow cytometry: comparison between instruments. Limnology and Oceanography—Methods.

[ref-64] Rivkin RB (1991). Seasonal patterns of planktonic production in Mcmurdo sound, Antarctica. American Zoologist.

[ref-65] Røstad A, Kaartvedt S, Aksnes DL (2016). Light comfort zones of mesopelagic acoustic scattering layers in two contrasting optical environments. Deep Sea Research Part I: Oceanographic Research Papers.

[ref-66] Sherr EB, Sherr BF, Longnecker K (2006). Distribution of bacterial abundance and cell-specific nucleic acid content in the Northeast Pacific Ocean. Deep Sea Research Part I: Oceanographic Research Papers.

[ref-67] Shibl AA, Haroon MF, Ngugi DK, Thompson LR, Stingl U (2016). Distribution of *prochlorococcus* ecotypes in the Red Sea basin based on analyses of rpoC1 sequences. Frontiers in Marine Science.

[ref-68] Silva L, Calleja ML, Huete-Stauffer TM, Ivetic S, Ansari MI, Viegas M, Morán XAG (2019). Low abundances but high growth rates of coastal heterotrophic bacteria in the Red Sea. Frontiers in Microbiology.

[ref-69] Sommer U (2000). Scarcity of medium-sized phytoplankton in the northern Red Sea explained by strong bottom-up and weak top-down control. Marine Ecology Progress Series.

[ref-70] Sommer U, Berninger UG, Böttger-Schnack R, Cornils A, Hagen W, Hansen T, Al-Najjar T, Post AF, Schnack-Schiel SB, Stibor H (2002). Grazing during early spring in the Gulf of Aqaba and the northern Red Sea. Marine Ecology Progress Series.

[ref-71] Tesfamichael D, Pauly D (2016). The Red Sea ecosystem and fisheries, Coral Reefs of the World.

[ref-72] Thompson LR, Williams GJ, Haroon MF, Shibl A, Larsen P, Shorenstein J, Knight R, Stingl U (2017). Metagenomic covariation along densely sampled environmental gradients in the Red Sea. ISME Journal.

[ref-73] Van den Engh GJ, Doggett JK, Thompson AW, Doblin MA, Gimpel CNG, Karl DM (2017). Dynamics of *prochlorococcus* and *synechococcus* at station ALOHA revealed through flow cytometry and high-resolution vertical sampling. Frontiers in Marine Science.

[ref-74] Waleron M, Waleron K, Vincent WF, Wilmotte A (2007). Allochthonous inputs of riverine picocyanobacteria to coastal waters in the Arctic Ocean. Fems Microbiology Ecology.

[ref-75] Wei YQ, Sun J, Zhang XD, Wang J, Huang K (2019). Picophytoplankton size and biomass around equatorial eastern Indian Ocean. Microbiologyopen.

[ref-76] Worden AZ, Nolan JK, Palenik B (2004). Assessing the dynamics and ecology of marine picophytoplankton: the importance of the eukaryotic component. Limnology and Oceanography.

[ref-77] Zhang Y, Jiao NZ, Hong N (2008). Comparative study of picoplankton biomass and community structure in different provinces from subarctic to subtropical oceans. Deep-Sea Research Part Ii-Topical Studies in Oceanography.

[ref-78] Zhu CS, Yu JM (2009). Nonmetric multidimensional scaling corrects for population structure in association mapping with different sample types. Genetics.

[ref-79] Zubkov M, Allen J, Fuchs B (2004). Coexistence of dominant groups in marine bacterioplankton community—a combination of experimental and modelling approaches. Journal of the Marine Biological Association of the United Kingdom.

[ref-80] Zubkov MV, Sleigh MA, Burkill PH, Leakey RJ (2000). Picoplankton community structure on the Atlantic Meridional Transect: a comparison between seasons. Progress in Oceanography.

[ref-81] Zwirglmaier K, Heywood JL, Chamberlain K, Woodward EM, Zubkov MV, Scanlan DJ (2007). Basin-scale distribution patterns of picocyanobacterial lineages in the Atlantic Ocean. Environmental Microbiology.

