# Supplementary material for: Seasonal variability and vertical distribution of autotrophic and heterotrophic picoplankton in the Central Red Sea
Source: PeerJ. 2020 Feb 24;8:e8612. doi: 10.7717/peerj.8612 (PMC7045887; doi:10.7717/peerj.8612)
Supplement: Figure S1 [file peerj-08-8612-s001.pdf]

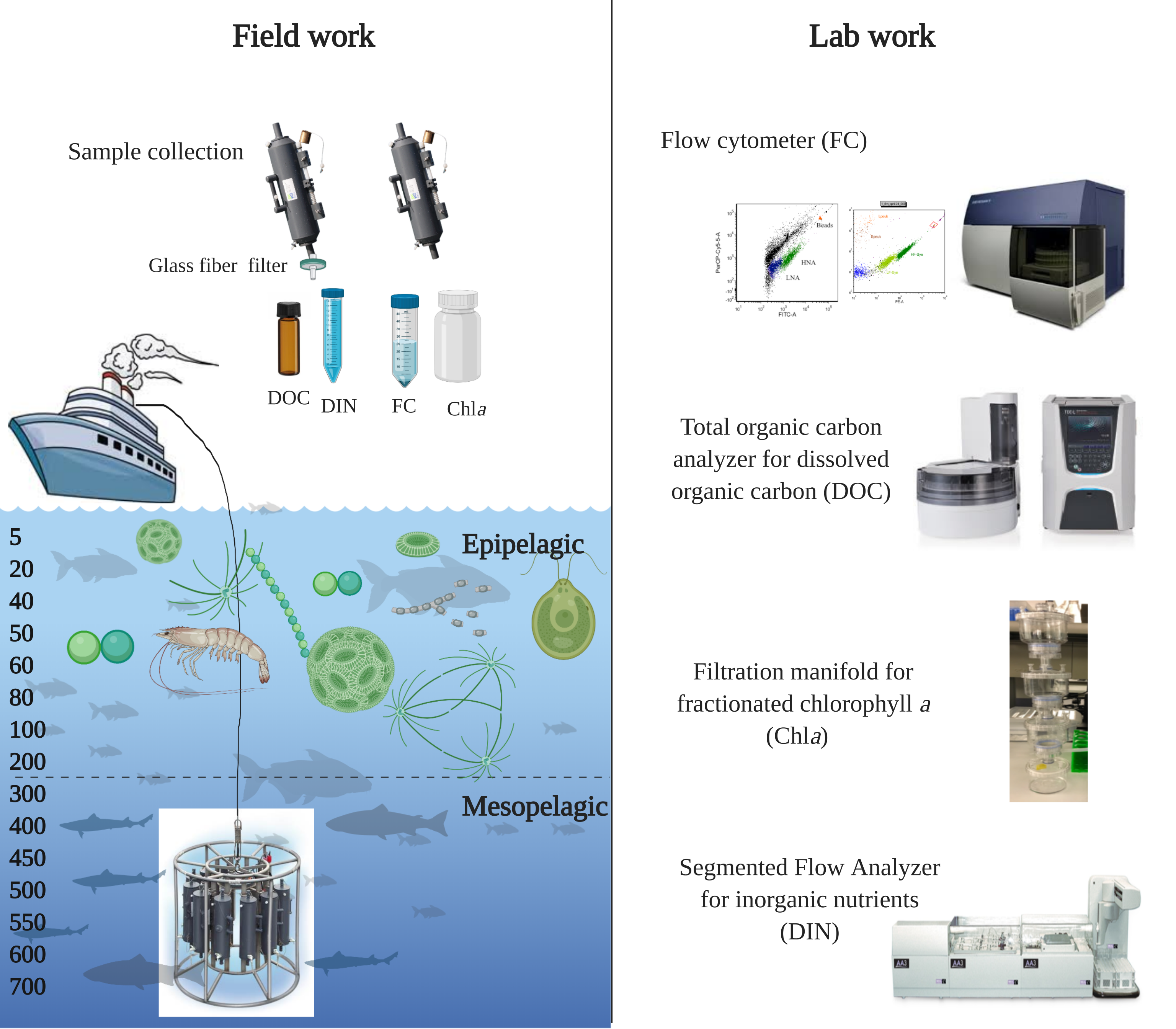
Field work
Lab work
Sample collection
Glass fiber filter
DOC
DIN
FC
Chla
5
20
40
50
60
80
100
200
300
400
450
500
550
600
700
Epipelagic
Mesopelagic
Flow cytometer (FC)
Beads
HNA
LNA
FITC-A
Total organic carbon analyzer for dissolved organic carbon (DOC)
Filtration manifold for fractionated chlorophyll a (Chla)
Segmented Flow Analyzer for inorganic nutrients (DIN)
