# Supplementary figures and images for: Seasonal variability and vertical distribution of autotrophic and heterotrophic picoplankton in the Central Red Sea
Source: PeerJ. 2020 Feb 24;8:e8612. doi: 10.7717/peerj.8612 (PMC7045887; doi:10.7717/peerj.8612)

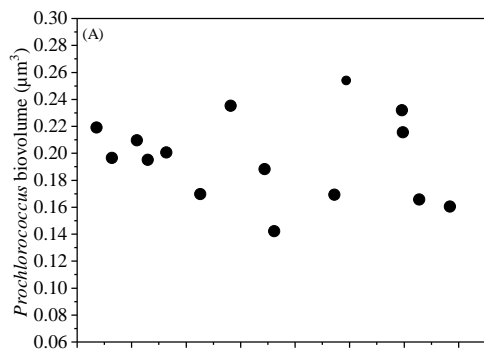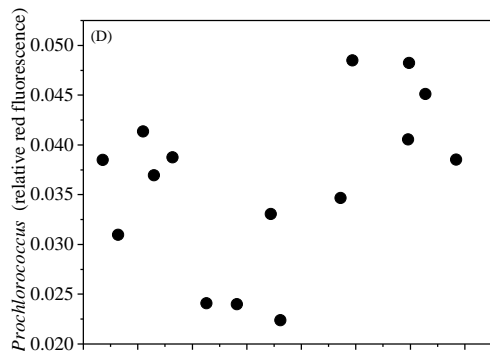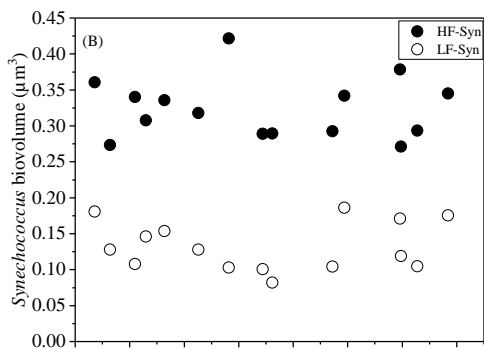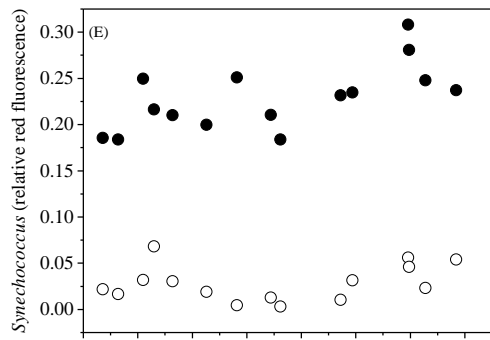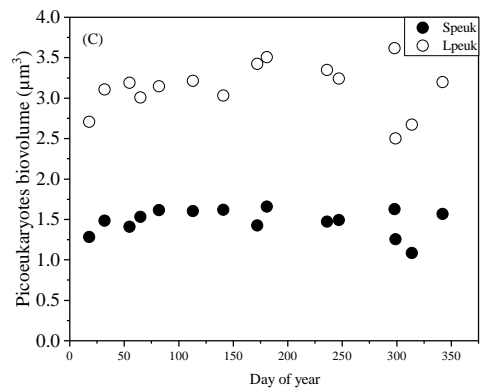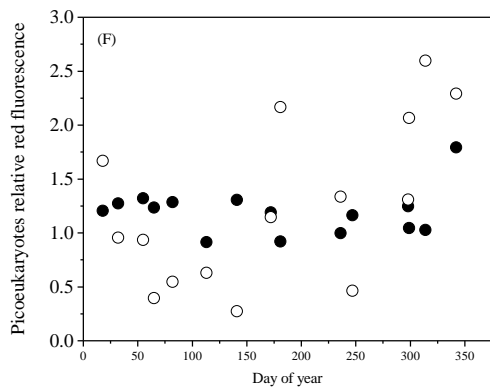

Supplement: Figure S2 — (A–C) biovolume and (D–F) relative red fluorescence of Prochlorococcus, high (HF-Syn) and low (LF-Syn) phycoerythrin fluorescence Synechococcus and small and large (Lpeuk) picoeukaryotes. [file peerj-08-8612-s002.pdf]

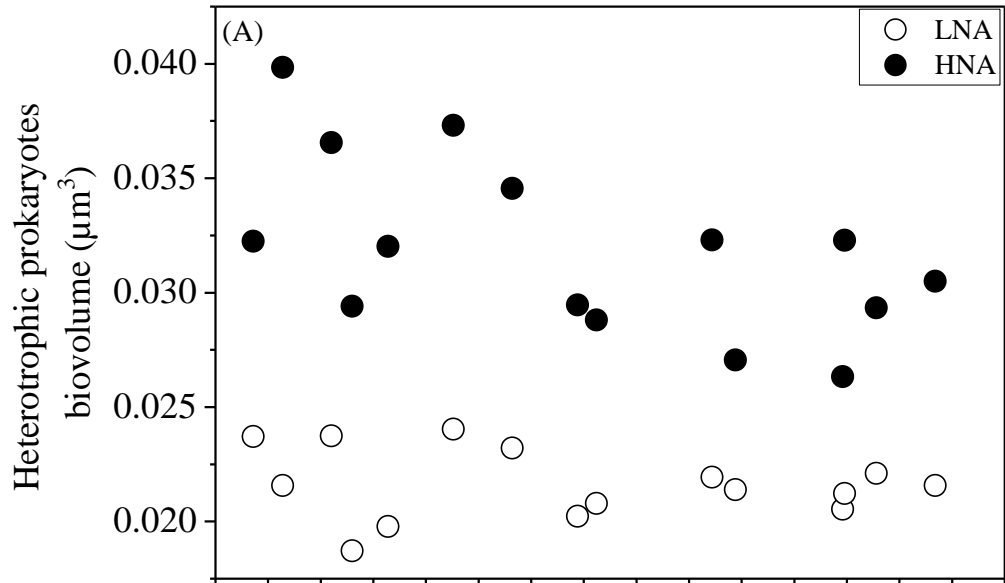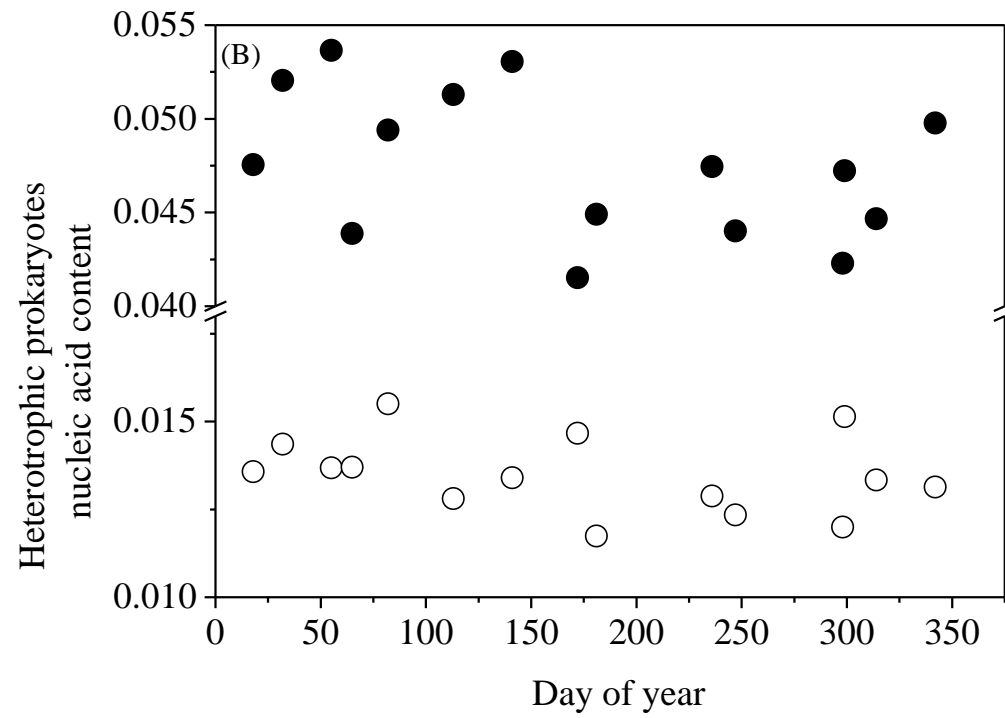

Supplement: Figure S3 — (A) biovolume and (B) nucleic acid content of low (LNA) and high (HNA) nucleic acid bacteria. [file peerj-08-8612-s003.pdf]

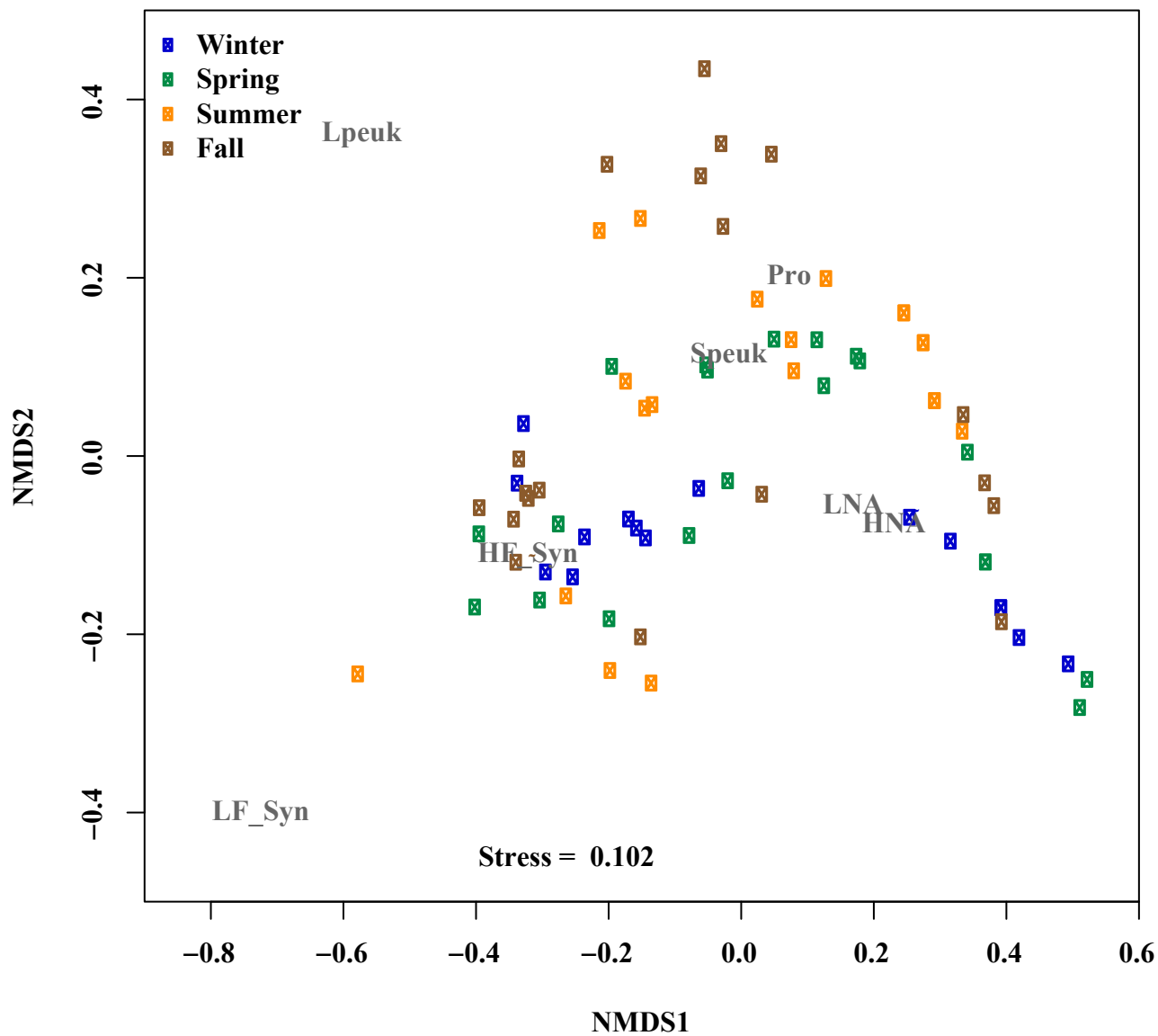

Supplement: Figure S4 — Autotrophic and heterotrophic picoplankton abbreviations as in Fig. 7 and in the main text. [file peerj-08-8612-s004.pdf]
